# The Effect of *Channa striatus* (Haruan) Extract on Pain and Wound Healing of Post-Lower Segment Caesarean Section Women

**DOI:** 10.1155/2015/849647

**Published:** 2015-05-25

**Authors:** Siti Zubaidah Ab Wahab, Azidah Abdul Kadir, Nik Hazlina Nik Hussain, Julia Omar, Rohaizan Yunus, Saringat Baie, Norhayati Mohd Noor, Intan Idiana Hassan, Wan Haslindawani Wan Mahmood, Asrenee Abd Razak, Wan Zahanim Wan Yusoff

**Affiliations:** ^1^School of Medical Sciences, Universiti Sains Malaysia, Health Campus, 16150 Kubang Kerian, Kelantan, Malaysia; ^2^School of Pharmaceutical Sciences, Universiti Sains Malaysia, 11800 Pulau Pinang, Malaysia; ^3^School of Health Sciences, Universiti Sains Malaysia, Health Campus, 16150 Kubang Kerian, Kelantan, Malaysia; ^4^Obstetrics & Gynaecology Department, Hospital Raja Perempuan Zainab II, 15586 Kota Bharu, Kelantan, Malaysia

## Abstract

*Channa striatus* has been consumed for decades as a remedy to promote wound healing by women during postpartum period. The objectives of this study were to compare postoperative pain, wound healing based on wound evaluation scale (WES), wound cosmetic appearance based on visual analogue scale (VAS) scores and patient satisfaction score (PSS), and safety profiles between *C. striatus* group and placebo group after six weeks of lower segment caesarean section (LSCS) delivery. A randomised, double-blind, placebo-controlled study was conducted. Subjects were randomised in a ratio of 1 : 1 into either the *C. striatus* group (500 mg daily) or placebo group (500 mg of maltodextrin daily). 76 subjects were successfully randomised, with 38 in the *C. striatus* group and 35 in the placebo group. There were no significant differences in postoperative pain (*p* = 0.814) and WES (*p* = 0.160) between the *C. striatus* and placebo groups. However, VAS and PSS in the *C. striatus* group were significantly better compared with the placebo group (*p* = 0.014 and *p* < 0.001, resp.). The safety profiles showed no significant differences between the groups. In conclusion, six-week supplementation of 500 mg of *C. striatus* extract showed marked differences in wound cosmetic appearance and patient's satisfaction and is safe for human consumption.

## 1. Introduction

*Channa striatus* is a fresh water snakehead fish, known in the Southeast Asian region for its protein and traditional remedy [1]. “Haruan” is the local name for* C. striatus *in Malaysia. It is a good source of health food amongst Asians because it contains high levels of amino acids and fatty acids [2].* C. striatus *is also normally consumed by women postpartum to promote wound healing as well as reduce postoperative pain [2, 3].

Many scientific studies have unravelled the biomedical potential of the fish. This species is known for having the beneficial aspects of anti-inflammatory, antimicrobial, antinociceptive, and anticancer properties [2, 4]. Studies have shown that* C. striatus *has a good antinociceptive property that makes it suitable for reduction of postoperative pain in a manner comparable to morphine [3].

In addition to those properties,* C. striatus *has been proven to enhance the wound healing process in animal studies [5, 6].* C. striatus *extract contains biochemical components, such as amino acids and fatty acids, which are important for the synthesis of collagen fibres during wound healing [1, 2], particularly glycine. This species also has high contents of arachidonic acid and polyunsaturated fatty acids that can promote prostaglandin synthesis [1, 2], which plays a vital role in healing the wounds [5, 6].

Thus, we undertook this study to assess the effects of* C. striatus *extract that may play possible roles or have great potential in wound healing and reducing postoperative pain in humans. This study was conducted to evaluate the effectiveness of* C. striatus *(Haruan) extract, as compared with a placebo, in terms of pain (postoperative pain), wound healing (wound evaluation scale), wound cosmetic appearance (visual analogue scale and patient's satisfaction score), and safety profiles (renal function test, liver function test, and full blood count) among post-lower segment caesarean section (LSCS) women.

## 2. Methodology

### 2.1. Study Design and Setting

This was a randomised, double-blind, two-arm parallel comparative study of* C. striatus *extract versus a placebo amongst women who have undergone LSCS at the Universiti Sains Malaysia Hospital (HUSM), Kubang Kerian, Kelantan, and Raja Perempuan Zainab II Hospital (HRPZ II), Kota Bharu, Kelantan, from May 2011 to January 2013.

The inclusion criteria were women aged between 18 and 40 years who had undergone a LSCS with no present active medical, surgical, or gynaecological problems. The exclusion criteria included women who had taken any form of herbal extract in the previous three months before study entry and during the study period, had a history of drug or alcohol abuse, used fresh* C. striatus *during the study period, were taking warfarin or heparin, had been diagnosed with clinically relevant cardiovascular, gastrointestinal, hepatic, neurologic, endocrine, hematologic, or connective tissue disease or another major systemic disease that would influence the interpretation of the results, had a medical disorder requiring steroid or immunosuppressive therapy, had a chronic cough or other condition that may cause an increase in intra-abdominal pressure, and showed any congenital anterior abdominal wall defects.

The orally administered freeze-dried* C. striatus *extract was prepared by a GMP-certified laboratory at the School of Pharmaceutical Sciences, Universiti Sains Malaysia. Both the freeze-dried* C. striatus *extract and maltodextrin were available in capsules of 250 mg. The treatment group consumed 500 mg of freeze-dried* C. striatus *extract, whereas the placebo group consumed 500 mg of maltodextrin. The capsules were taken orally with water once daily and at any time of the day, with or without a meal.

Informed consent was obtained from the women 24–48 hours after the elective or emergency LSCS. The researchers ensured that the women were fully conscious and comfortable before informed consent was taken. Information on demographic data and past and concurrent medical history was obtained by interviewing the women. The researcher then performed physical examinations on the women.

On day 3 after the operation, the women were assessed for postoperative pain based on the numeric pain rating scale (NRS), wound healing based on the WES, wound cosmetic appearance based on the VAS, and patients' satisfaction regarding wound appearance based on patient satisfaction scores (PSS). The subjects were subsequently allocated into two groups using a computer-generated randomisation of numbers. The women were followed up at postoperative week 2, week 4, and week 6 for postoperative pain, wound healing, wound appearance, and patient satisfaction of the wound appearance. At every visit, any adverse event (AE) and concomitant medications were recorded. In addition, the women's compliance was measured at every visit using the number of capsules taken. Assessment of liver function, renal function, and full blood count was conducted at baseline and at the last visit (postoperative week 6) for measurement of the safety profiles. In this study, all the women underwent standard procedures for the LSCS and received standard postoperative pain management.

### 2.2. Outcome Measures

The outcome measures used in this study were 0–10 NRS [7] for postoperative pain, WES [8, 9] for wound healing, VAS [10] for wound cosmetic appearance, and PSS for overall patient's satisfaction with the appearance of the wound [11].

The WES was first developed by Hollander et al. [8] and later modified by Quinn and Wells [9], who proved that WES was a reliable tool in assessing surgical wounds. It consists of six items: presence or absence of wound discharge, edge inversion, step-off borders, contour irregularities, scar width more than 2 mm, and overall cosmesis. Each of these categories was graded on a 0-point (*presence*) or 1-point (*absence*) scale. A total cosmetic score was derived by adding the scores of the six categorical variables.

The VAS has been used globally in numerous cosmetic studies [12–14]. This scale consisted of a 100 mm line signifying the “worst scar” at the far right end and the “best scar” at the far left end of the line. The higher the score was, the better the cosmetic appearance was. Meanwhile, PSS was rated based on scores of 0 to 10, with higher scores indicating better satisfaction and lower scores indicating poorer satisfaction. The PSS has been validated by Coulthard et al. [11] and Blondeel et al. [15] and revised by Amin et al. [16].

### 2.3. Sample Size Calculation

Sample size was calculated for all objectives of the study and the biggest sample size calculated was taken. The calculations were done using Power and Sample Size Calculation Software [17] for comparing two means between treatment group and placebo group. The biggest sample size calculated was yielded by wound healing assessment based on visual analogue scale. By using a standard deviation of 19 [9], taking the power of 90%, detectable difference of 15 [9], and level of significance of 0.05, the calculated sample size for each group was 35. However after considering 10% dropout, the sample size for each group is 38.

### 2.4. Statistical Analysis

Data were analyzed by SPSS version 20. In this study, the subjects were analyzed based on their assigned group, regardless of intervention attendance or compliance (intention-to-treat analysis). According to the protocol, the last-observation-carried-forward approach was used for patients who did not complete the study. Repeated measures for analysis of variance (RM ANOVA) were used to compare postoperative pain scores and PSS at baseline, week 2, week 4, and week 6. Body mass index (BMI) was added as a controlled variable for the analysis of covariance- (ANCOVA-) RM for WES and VAS. All *p* values were reported as two-tailed results. The level of statistical significance was set at 0.05.

### 2.5. Approval by the Ethical Committee

Ethical permission was obtained from the ethics research committee (Human) of the Universiti Sains Malaysia and with the permission of the Director of the HUSM on 31 December 2009 (Ref: USMKK/PPP/JEPeM [220.3.(04)]). For the HRPZ II, approval from the National Medical Research Register (NMRR) [Ref: NMRR-11-1018-10092] was obtained.

## 3. Results

**Figure 1:**
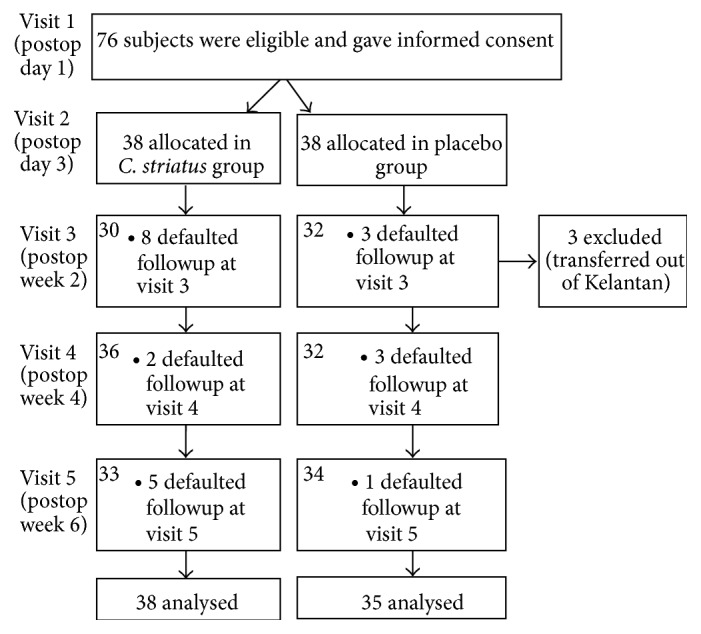
Flowchart of subject's progress throughout the study.

Seventy-six subjects were successfully randomised into this study. The details are shown in Figure 1. Thirty-eight subjects were allocated to the* C. striatus *group, and thirty-eight subjects were allocated to the placebo group.

### 3.1. Baseline Characteristics of the Subjects

The demographic distribution and baseline clinical measurements of the subjects are shown in Tables 1 and 2. There was no significant difference detected between the* C. striatus *and placebo groups in terms of age, parity, and type of LSCS. However, there was a significant difference in terms of BMI between the* C. striatus *and placebo groups.

### 3.2. Intergroup Differences Based on Time (Postoperative Pain, WES, VAS, and PSS)

Table 3 shows the intergroup differences of NRS, WES, VAS, and PSS based on time. The VAS and PSS scores were significantly higher in the* C. striatus *group than in the placebo group (*F* = 3.78, *p* = 0.014, and *F* = 9.06, *p* < 0.001). This difference suggests that the* C. striatus *group had better wound appearance and higher PSS than the placebo group.

The* C. striatus *group showed a larger decrease in pain score over time compared with the placebo group, but this difference was not statistically significant (*F* = 0.32, *p* = 0.814). The same trends were observed for the WES, in which the scores were higher in the* C. striatus *group compared with the placebo group, but this finding was also not statistically significant (*F* = 1.78, *p* = 0.160).

### 3.3. Safety Profiles

There were no AEs reported by study subjects. All physical examinations and vital signs were within normal limits. Compliance to medication was 98–100%. Tables 4 and 5 represent the lack of significant differences in all safety parameters between the* C. striatus *and placebo groups at baseline (*p* > 0.05) and the study endpoint (week 6; *p* > 0.05).

## 4. Discussion

In this study, we found that* C. striatus *extract could improve the cosmetic appearance of wounds and achieve high patient satisfaction regarding the cosmetic appearance of the wound. The VAS has been proven to have the highest sensitivity in detecting differences in the cosmetic appearance of a surgical wound [10]. The VAS has been used worldwide in cosmetics involving various types of wounds, such as reconstructive plastic surgery [13], laparoscopic pyeloplasty [14], scalp wounds [18], breast cancer surgery [19], breast augmentation surgery [12], and paediatric facial laceration wounds [20]. The VAS is usually used as an adjuvant assessment of other wound assessment tools, such as the patient and observer scar assessment scale, Vancouver scar scale, ASEPSIS, surgeon-assessed Hollander scale, and the Manchester scar scale [19, 21, 22]. In the current study, VAS was used in adjuvant of WES.

There are a few plausible explanations behind these positive findings. The effectiveness of* C. striatus *as a wound cosmetic enhancer is thought to be influenced by the high level of specific amino acids, such as glycine, and fatty acids, such as arachidonic acid, in its makeup [5, 6]. These two compounds were believed to be involved in the promotion of wound healing by the initiation of a series of mechanisms involving the remodelling of collagen, reepithelialisation of the wound, and induction of wound contraction [5, 6]. Glycine is important in the healing process when combined with arachidonic acid [2]. These acids are part of the major components of human skin collagen, which, when acting synergistically with other essential amino acids such as proline, alanine, arginine, isoleucine, phenylalanine, and serine, form a polypeptide that promotes tissue repairs and healing [23, 24].

*C. striatus *extract also contains arginine [2]. Barbul et al. [25] showed that arginine supplementation significantly enhanced the amount of collagen deposited into a standardised wound, as assessed by the larger amount of hydroxyproline present (*p* < 0.001). These effects also may have contributed to the improvement of the cosmetic appearance and PSS in the current study. Mustafa et al. (2012) [26] reported that* C. striatus *extract contains 3.43 ± 0.28 mg/100 mL of zinc (Zn). Administration of Zn orally could accelerate the healing process of surgical wounds [26]. This benefit could be because Zn plays important roles in protein synthesis and cell multiplication.

The interval of two weeks for followup in this study is the main factor for the insignificant results of pain assessment and wound assessment based on WES. For the postoperative pain score, women's pain levels were adequately controlled because they had received standard postoperative pain management. This management was evidenced by the mean scores of both groups for pain, which were approximately 2-3, indicating that the pain levels were mild. Hence, for postoperative pain in this study, we conclude that the interval between assessment of pain and the use of concurrent analgesics influenced this study's insignificant results.

However, in an animal study conducted by Mat Jais et al. [3], the tail flick test (heat stimulus) and abdominal constriction test (chemical stimulus) showed that* C. striatus *extract does act as a potent analgesic agent alone and enhances other analgesics, such as morphine. It should be noted that the administration of these tests in that study was performed intraperitoneally, which differs from the oral administration of* C. striatus *extract in this study.

The same explanation goes for the insignificant result of the wound assessment based on the WES score. The first wound assessments were performed on postoperative day 3, in which there was still the presence of significant inflammation. Within the first 14 days, it was assumed that the entire wound had healed and the process of scarring was in progress. The WES score yielded insignificant results because of the normal healing process that was missed to assess within the first two weeks.

Ideally, the best way to assess wound healing clinically is to monitor the wound as closely as possible each day. However, it is not feasible and practical to prolong the hospital stay of the women just for wound observation. Self-photographs of the wound every day are another option, but the photograph might not be standardised in terms of lightning, position, and sharpness, which could lead to a range of biases. In this study,* C. striatus *extract did not present any AEs on the haematological profile. This finding was in line with a number of studies that proved natural products are safe to be consumed by humans [27–30].

There were several limitations identified in this study. For instance, a major limitation was the defaulter for the followup in this study. In addition, we struggled to make the duration of followup shorter to get better results. Future clinical trials could use a different cohort of surgical subjects, such as postappendectomy patients or those with bone fractures, to allow for closer daily monitoring of the wound and better determination of the percentage of wound healing. The trial of other formulations of the extract, such as cream or spray applications, for human wound healing would also be useful. Future works should further emphasize finding bioactive compounds and research on standardisation of the extract.

## 5. Conclusion

From the findings of this study, it can be concluded that the oral administration of* C. striatus *extract 500 mg daily does have marked differences in terms of wound cosmetic appearance and patient satisfaction score towards the wound appearance. However, this extract does not show any significant reduction of postoperative pain and improvement of wound healing based on the WES. The oral administration of* C. striatus *extract at 500 mg daily did not produce any AEs to the study subjects.

## Figures and Tables

**Table 1:** Demographic characteristic of the subjects.

Demographic characteristic	*C. striatus *(*n* = 38)		Placebo (*n* = 35)		*p* value
	Mean (SD)	*n* (%)	Mean (SD)	*n* (%)	
Age	28.11 (4.93)		28.83 (5.90)		0.570^*∗*^
Parity	2.42 (1.69)		2.09 (1.69)		0.399^*∗*^
BMI	23.06 (3.50)		25.79 (5.63)		0.014^*∗*^
Types of LSCS					
Elective		1 (2.6)		5 (14.3)	0.875^*∗∗*^
Emergency		37 (97.4)		30 (85.7)	

^*∗*^Determined by independent *t*-test.

^*∗∗*^Determined by simple logistic regression.

BMI = body mass index; LSCS = lower segment caesarean section.

**Table 2:** Clinical characteristics of study subjects at baseline (postoperative day 3).

Clinical characteristic	*C. striatus *(*n* = 38)	Placebo (*n* = 35)	*p* value
	Mean (SD)	Mean (SD)	
Postoperative pain score	3.05 (1.36)	3.37 (1.26)	0.303
Wound evaluation scale (WES)	4.89 (0.31)	4.89 (0.32)	0.904
Visual analogue scale (VAS)	5.87 (0.34)	5.86 (0.36)	0.891
Patient's satisfaction score (PSS)	5.37 (2.26)	5.40 (2.08)	0.951

*p* values were determined using independent *t*-tests.

**Table 3:** Intergroup differences based on time for postoperative pain, WES, VAS, and PSS.

Outcomes	Group	Postop day 3	Postop week 2	Postop week 4	Postop week 6	*F*-statistic	*p* value
		Mean (95% CI)	Mean (95% CI)	Mean (95% CI)	Mean (95% CI)		
Postoperative pain score	*C. striatus *	3.05 (2.63, 3.48)	2.05 (1.51, 2.60)	1.03 (1.41, 2.65)	0.45 (0.11, 0.78)	0.32	0.814^*∗*^
	Placebo	3.37 (2.93, 3.81)	2.17 (1.60, 2.74)	1.46 (1.06, 1.85)	0.66 (0.31, 1.00)		

Wound evaluation scale (WES)	*C. striatus *	4.89 (4.79, 5.00)	5.47 (5.14, 5.79)	5.36 (4.95, 5.77)	5.43 (5.05, 5.80)	1.78	0.160^*∗∗*^
	Placebo	4.89 (4.78, 5.00)	5.44 (5.10, 5.78)	4.93 (4.49, 5.36)	5.17 (4.78, 5.56)		

Visual analogue scale (VAS)	*C. striatus *	5.86 (5.74, 5.97)	5.86 (5.46, 6.25)	6.85 (6.41, 7.28)	7.24 (6.72, 7.76)	3.78	0.014^*∗∗*^
	Placebo	5.87 (5.75, 6.00)	5.67 (5.26, 6.08)	6.05 (5.60, 6.51)	6.71 (6.17, 7.26)		

Patient's satisfaction (PSS)	*C. striatus *	5.37 (4.64, 6.09)	7.78 (7.22, 8.35)	9.89 (9.59, 10.00)	9.74 (9.53, 9.96)	9.06	<0.001^*∗*^
	Placebo	5.40 (4.65, 6.16)	7.58 (6.99, 8.17)	8.64 (8.32, 8.95)	9.54 (9.31, 9.76)		

^*∗*^RM ANOVA was applied, followed by pairwise comparison with Bonferroni confidence interval adjustment. Assumption of normality, homogeneity of variances, and compound symmetry were checked and fulfilled.

^*∗∗*^RM ANCOVA was applied, followed by pairwise comparison with Bonferroni confidence interval adjustment. Numerical covariate (BMI) was controlled. Assumption of normality, homogeneity of variances, and compound symmetry were checked and fulfilled.

**Table 4:** Baseline (postop day 3) safety parameters.

Parameters	*C. striatus *(*n* = 38)	Placebo (*n* = 35)	*p* value
	Mean (SD)	Mean (SD)	
Urea (mmol/L)	4.15 (1.19)	4.21 (1.38)	0.847
Creatinine (*µ*mol/L)	73.82 (46.02)	73.94 (10.17)	0.987
Total protein (g/L)	66.02 (33.61)	64.00 (21.20)	0.543
Albumin (g/L)	32.33 (12.14)	34.30 (13.11)	0.511
Aspartate aminotransferase (U/L)	23.74 (11.98)	25.54 (15.70)	0.581
Alanine transaminase (U/L)	15.29 (7.34)	15.94 (9.52)	0.742
White blood cell count (×10^9^/L)	14.10 (2.73)	12.61 (3.23)	0.505
Haemoglobin (g/L)	10.38 (1.77)	11.03 (2.22)	0.337
Platelet (×10^9^/L)	247.92 (57.75)	258.26 (49.40)	0.416

*p* values were determined using independent *t*-tests.

**Table 5:** Study endpoint (week 6) safety parameters.

Parameters	*C. striatus *(*n* = 38)	Placebo (*n* = 35)	*p* value
	Mean (SD)	Mean (SD)	
Urea (mmol/L)	4.48 (1.21)	4.34 (1.18)	0.130
Creatinine (*µ*mol/L)	78.11 (8.17)	79.43 (9.38)	0.875
Total protein (g/L)	80.14 (24.25)	84.65 (26.34)	0.214
Albumin (g/L)	38.23 (8.54)	37.06 (6.12)	0.538
Aspartate aminotransferase (U/L)	20.43 (6.71)	20.37 (6.92)	0.817
Alanine transaminase (U/L)	17.87 (8.79)	17.57 (16.58)	0.896
White blood cell count (×10^9^/L)	6.76 (1.43)	7.14 (1.66)	0.295
Haemoglobin (g/L)	11.50 (2.61)	11.55 (2.37)	0.337
Platelet count (×10^9^/L)	277.39 (48.21)	293.00 (71.99)	0.277

*p* values were determined using independent *t*-tests.
